# A high-throughput genome-wide siRNA screen for ciliogenesis identifies new ciliary functional components and ciliopathy genes

**DOI:** 10.1186/2046-2530-4-S1-O12

**Published:** 2015-07-13

**Authors:** K Szymanska, G Wheway, D Doherty, M Schmidts, D Mans, TMT Nguyen, K Boldt, G Tödt, Z Abdelhamed, K Wunderlich, S Natarajan, DA Parry, CV Logan, W Herridge, N Sorusch, U Wolfrum, M Ueffing, R Roepman, H Mitchison, C Johnson

**Affiliations:** 1 Ophthalmology and Neuroscience, University of Leeds, Leeds, UK; 2 Divisions of Developmental Medicine and Genetic Medicine, University of Washington, Seattle, USA; 3 Molecular Medicine Unit and Birth Defect Research Center, University College London, London, UK; 4 Department of Human Genetics, Radboud University Medical Centre, Nijmegen, The Netherlands; 5 Division of Experimental Ophthalmology and Medical Proteome Center, University of Tübingen, Tübingen, Germany; 6 Structural and Computational Biology, European Molecular Biology Laboratory, Heidelberg, Germany; 7 Department of Cell and Matrix Biology, Johannes Gutenberg University of Mainz, Mainz, Germany; 8 Section of Genetics, University of Leeds, Leeds, UK

## 

Defects in primary cilium biogenesis underlie the ciliopathies, a growing group of genetic disorders. We describe the first whole genome siRNA-based reverse genetics screen for defects in biogenesis and/or maintenance of the primary cilium, obtaining a global resource for investigation and interventions into the processes that are critical for the ciliary system. In total, we identified 83 candidate ciliogenesis and ciliopathy genes, including 15 components of the ubiquitin-proteasome system. The validated hits also include 12 encoding G-protein-coupled receptors, and three encoding pre-mRNA processing factors (PRPF6, PRPF8 and PRPF31) mutated in autosomal dominant *retinitis pigmentosa*. Combining the screen with exome sequencing data identified recessive mutations in screen candidate genes as novel causes of ciliopathies, emphasizing the utility of our screen for ciliopathy gene discovery. Our findings emphasize the relevance of global, unbiased functional and genetic screening approaches in understanding ciliogenesis complexity, and in identifying loss of function in unanticipated pathways of human genetic disease.

